# Virulence test using nematodes to prescreen *Nocardia* species capable of inducing neurodegeneration and behavioral disorders

**DOI:** 10.7717/peerj.3823

**Published:** 2017-10-10

**Authors:** Claire Bernardin Souibgui, Anthony Zoropogui, Jeremy Voisin, Sebastien Ribun, Valentin Vasselon, Petar Pujic, Veronica Rodriguez-Nava, Patrick Belly, Benoit Cournoyer, Didier Blaha

**Affiliations:** 1 UMR CNRS5557, INRA1418 Ecologie Microbienne, Université Lyon 1, VetAgro Sup, Université Claude Bernard (Lyon I), Lyon, France; 2 Department of Clinical and Morphological Pathology, Université de Lyon, VetAgro Sup Campus Vétérinaire de Lyon, Marcy L’Etoile, France, Université Claude Bernard (Lyon I), France; 3 Université Claude Bernard (Lyon I), France

**Keywords:** *Nocardia*, *C. elegans*, Parkinson’s symptoms, Neuronal apoptosis, Rapid virulence test

## Abstract

**Background:**

Parkinson’s disease (PD) is a disorder characterized by dopaminergic neuron programmed cell death. The etiology of PD remains uncertain—some cases are due to selected genes associated with familial heredity, others are due to environmental exposure to toxic components, but over 90% of cases have a sporadic origin.* Nocardia* are Actinobacteria that can cause human diseases like nocardiosis. This illness can lead to lung infection or central nervous system (CNS) invasion in both immunocompromised and immunocompetent individuals. The main species involved in CNS are *N. farcinica, N. nova*, *N. brasiliensis* and *N. cyriacigeorgica*. Some studies have highlighted the ability of *N. cyriacigeorgica* to induce Parkinson’s disease-like symptoms in animals. Actinobacteria are known to produce a large variety of secondary metabolites, some of which can be neurotoxic. We hypothesized that neurotoxic secondary metabolite production and the onset of PD-like symptoms in animals could be linked.

**Methods:**

Here we used a method to screen bacteria that could induce dopaminergic neurodegeneration before performing mouse experiments.

**Results:**

The nematode *Caenorhabditis elegans* allowed us to demonstrate that *Nocardia* strains belonging to *N. cyriacigeorgica* and *N. farcinica* species can induce dopaminergic neurodegeneration. Strains of interest involved with the nematodes in neurodegenerative disorders were then injected in mice. Infected mice had behavioral disorders that may be related to neuronal damage, thus confirming the ability of *Nocardia* strains to induce neurodegeneration. These behavioral disorders were induced by *N. cyriacigeorgica* species (*N. cyriacigeorgica* GUH-2 and *N. cyriacigeorgica* 44484) and *N. farcinica* 10152.

**Discussion:**

We conclude that *C. elegans* is a good model for detecting *Nocardia* strains involved in neurodegeneration. This model allowed us to detect bacteria with high neurodegenerative effects and which should be studied in mice to characterize the induced behavioral disorders and bacterial dissemination.

## Introduction

Parkinson’s disease (PD) is the second most frequent neurodegenerative disorder after Alzheimer’s disease. With the rise in the population mean age, the prevalence of this illness is increasing, affecting millions of individuals worldwide. PD is a slowly evolving disorder characterized by bradykinesia, rigidity, tremor and postural instability. The pathological hallmark of PD is the degeneration of dopaminergic neurons localized in the *substancia nigra pars compacta*, resulting in loss of the nigrostriatal pathway and a reduction of dopamine levels in the striatum (Braak et al., 2003). For many years, PD was considered a nongenetic disorder caused by synergistic environmental factors. Large genome-wide association studies (GWAS) have identified more than two dozen common genetic variants for PD, each with a relatively small effect size; in combination with rare Mendelian genes, genetics account for at best 10–20% of PD (Lill et al., 2012; Nalls et al., 2014; Trinh & Farrer, 2013). The majority of PD cases have a sporadic origin, and the environment seems to have a critical impact on the epidemiology of this illness (Goldman, 2014; Ritz, Paul & Bronstein, 2016). Several studies have suggested that rural environments may be epidemiological contributors to PD. It is well known that pesticides and herbicides like rotenone, paraquat, and MPTP are etiologic agents of PD (Hatcher, Pennell & Miller, 2008; Khandhar & Marks, 2007). Indeed, these molecules are lipophilic—they are able to cross the blood-brain barrier, the neuronal cellular membrane and cause oxidative stress, in turn inducing neurodegeneration. Animal models of PD involving these pesticides have been developed by several research teams. The action of these toxins was noted, and a dysfunction in the ubiquitin-proteasome system (UPS) involved in protein degradation has also been frequently observed. Toxins that can inhibit the UPS have been identified as secondary metabolites produced by microorganisms. For instance, proteasome inhibitors like epoxomicin and lactacystin can cause impairment of the UPS responsible for neurodegeneration in animal models (McNaught et al., 2004; Salama & Arias-Carrión, 2011).

*Nocardia* are aerobic Gram-positive actinomycetes bacteria with a high G + C percentage. They are important components of the soil microbiome and can also be found in fresh and salt water environments (Brown-Elliott et al., 2006; Wilson, 2012). Until now, more than 80 *Nocardia* species have been described in the literature, with 33 being responsible for human diseases (Abreu et al., 2015; Brown-Elliott et al., 2006; Wilson, 2012). These bacteria can be aerosolized in dust, which can be inhaled (Ambrosioni, Lew & Garbino, 2010; Brown-Elliott et al., 2006) and lead to lung infections. The central nervous system is the second most commonly infected organ by *Nocardia* spp. (Beaman et al., 1976; Ogata & Beaman, 1992). In humans, cerebral nocardiosis may cause the following symptoms: nausea, vomiting with photophobia, headache, neck stiffness, motor disorders (hemiparesis and tremors) and behavioral disorders (schizophrenia, depression, dyslexia, hallucinations and amnesia) (Beaman & Beaman, 1994). Kohbata & Beaman (1991) reported that a sublethal injection of *Nocardia cyriacigeorgica* GUH-2 can induce a syndrome in mice which shares clinical and pathological similarities with PD. These results were confirmed in other studies (Kohbata & Beaman, 1991; Ogata & Beaman, 1992). *Streptomyces venezuale*, another actinomycete, was also described as being able to produce secondary metabolites which could induce dopaminergic neurodegeneration (Caldwell et al., 2009).

**Figure 1:**
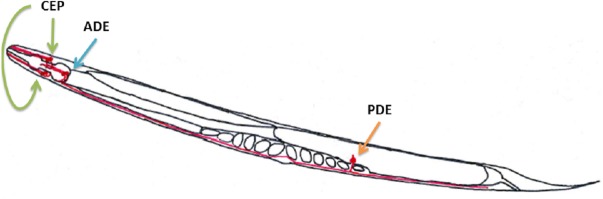
Dopaminergic neuron locations in *C. elegans* according to the WormAltas. The neuronal body and the axons are shown in red. The green arrows indicate the four CEP neurons, the blue ones indicate the two ADE neurons and the orange ones indicate the PDE neurons. Only one PDE neuron is represented because the other one was behind the organs.

The aim of this study was to develop a method to study *Nocardia* properties involved in neuronal virulence and assess the health risks that various *Nocardia* species isolated from clinical and environmental samples may represent. This test was designed so that the number of isolates analyzed would be higher than in the mouse model. This method consists of performing a test on the nematode *C. elegans* that was previously described as a good model for studying neurotoxicity induced by *S. venezuelae* (Caldwell et al., 2009; Harrington et al., 2010; Martinez, Caldwell & Caldwell, 2017). *C. elegans* has 302 neurons, eight of which are dopaminergic neurons. These dopaminergic neurons are located in the nematode as follows: (i) six are in the anterior part of the nematode and consist of two pairs of cephalic neurons (CEP) and one pair of *class E* anterior deirid neurons (ADE); and (ii) two *class E* posterior deirid neurons (PDE) in the posterior part of the animal (Fig. 1) (Berkowitz et al., 2008; Locke et al., 2008). Modifications in these structures may indicate a neurotoxic effect of the bacterial supernatant.

## Materials and methods

### *Nocardia* strains

Table 1 indicates the *Nocardia* strains used in this study. *Nocardia* strains were grown at 37 °C shaking at 150 rpm in BHI-P medium (for BALB/c mouse experiments) and in Bennett liquid medium (for nematode tests) because BHI-P medium was toxic *C. elegans*. Then, for tests on nematodes, culture supernatants were recovered after a one month incubation period for *N. farcinica* IFM 10152 and two months for *N. cyriacigeorgica* and *N. asteroides* strains. These conditions were defined according results obtained in preliminary tests. For the BALB/c mouse tests, *Nocardia* cells were grown in order to recover 3, 5 × 10^5^ CFU mL^−1^.

**Table 1:** *Nocardia* strains used in this study. Seven strains from different origins (clinical or environmental) were used in this study. Strains tested on mice and nematodes are indicated.

Strains	Origin	Mouse experiment	Nematode experiment	Reference
*N. cyriacigeorgica* DSM 44484	Clinical	+	+	Yassin, Rainey & Steiner (2001)
*N. cyriacigeorgica* OFN 04.100	Clinical		+	OFN’s collection
*N. cyriacigeorgica* OFN 04.107	Clinical		+	OFN’s collection
*N. cyriacigeorgica* GUH-2	Clinical	+	+	Beaman & Maslan (1978)
*N. cyriacigeorgica* OFN N27	Environmental		+	OFN collection
*N. farcinica* IFM 10152	Clinical	+	+	Ishikawa et al. (2004)
*N. asteroides* ATCC19247	Environmental	+	+	Gordon & Mihm (1959)

### Nematode neurodegeneration assay

The *C. elegans* BY250 *vtIs* 7 [Pdat-1:GFP] line was used. This is a transgenic line specifically expressing GFP in dopaminergic neurons (*dat-1* promotor) (Kohbata & Shimokawa, 1993). The integrity of the six anterior dopaminergic neurons was monitored with this *C. elegans* line. In our experiments, *C. elegans* strains were cultured on NGM medium and fed with *E. coli* OP50 at 23 °C according to standard methods (Brenner, 1974; Hope, 1999). Gravid nematodes were dropped onto plates and removed around 6 h later, leaving time for egg laying. Eggs were then incubated for 3 days at 15 °C. Nematodes at the L4 development stage were then picked up and dropped onto NGM medium supplemented with 10 µM 5-FU (5-fluorouracil). The same experiment was done without 5-fluorouracil and we obtained different nematode development stages. This variability had an effect on their neuronal viability, probably due to their age. 5-FU was thus used to block the development of new eggs in order to standardize the assay. This step represented day 0 of the experiment. Nematodes were plated with filtered Bennett supernatants recovered from *Nocardia* broth. Supernatants were recovered from the first plating of nematodes (egg-laying period) and then at days 0, 2 and 4 at 23 °C. Some nematodes where exposed to sterile Bennett broth for control. At day 6, for each bacterial supernatant tested, 30 nematodes per condition were placed on 2% agarose pads, fixed with tetramisol (5 mM) and observed by fluorescence microscopy with a GFP38He filter. Microscopic analyses were performed with an Axio imager.Z1 (Zeiss). Nematodes were considered as having a wild-type phenotype when they showed no neuronal abnormalities. Nematodes with dendrite blebbing or beading, neuronal cell body rounding, or cell body and/or process loss were considered as affected. Blebbing and beading are different modifications along the axonal process. Blebbing can be defined as triangular-shaped protrusions, and beading as focal enlargements, but here we do not differentiate these two terms, and use the generic term “blebbing” for both phenomena (Chew et al., 2013). Behavioral tests for dopamine function were performed using: (i) a touching test on nematodes, and (ii) body-bend counting (one body bend is deemed as one sinusoidal movement until the worm reaches the same posture again). The first test consists of touching the nose of the nematodes and in observing their behavioral reactions. The second involves counting body-bends per minute for 20 nematodes per condition (Taferner et al., 2015; Yu & Liao, 2014). The wild-type *C. elegans* line (N2) and a transgenic line (BY250) was used for this behavior test.

### BALB/c experiments

Female BALB/c mice weighing 18–20 g were used, and handled in a level 2 safety lab at Claude Bourgelat Institute® (Vetagro Sup, Marcy l’Etoile, France). ISOcages ™ were used for this experiment. Animals were acclimated for 10 days to their environment prior to testing. All experiments were approved by the VetAgro Sup ethics committee (authorization number 722). Each BALB/c mouse received a sublethal injection of *Nocardia* (around 3, 5 × 10^5^ CFU mL^−1^) through the lateral tail vein, as described by Kohbata & Beaman (1991). Behavioral disorders in mice were observed 13 days after infection. The behavioral disorders were: hemiparesis, muscular rigidity, tremors throughout the body or vertical head movements. Mice selected for anatomo-pathology analyses were those having the most severe symptoms. BALB/c mice were euthanized at the end of the experiments, after anesthesia (intraperitoneal injection of ketamine (100 mg kg^−1^)), by an intraperitoneal injection of 0.5 ml of a dolethal solution. Some organs were collected. Brains were cut to separate the two hemispheres. The first part was frozen in liquid nitrogen and conserved at −80 °C, the second part was immersed in histological buffered formalin (pH 7.4–7.6), for further analyses. After fixation in histological buffered formalin, organs were dehydrated using five successive ethanol baths (first 70°, second 90° and third close to absolute ethanol) and then were introduced in three butyl ethanol baths. Finally, samples were immersed in a paraffin bath at 60 °C. Serial section 4 µm thick were cut from the paraffin organ blocks. Each series of six cups were done every 400–500 µm to be representative of the entire organ. Each series was stained differently: Harris-eosin hematoxylin stain, Fite stain, Gram stain, histochemical and immunochemical stain. Rabbit anti-mycobacterium polyclonal antibody (SEROTEC OBT0947) was used for histochemical and immunochemical staining.

### Statistical tests

Statistical tests were performed with the R v.2.14.0 package (R Core Team, 2011). Fisher exact tests were performed between strains and controls in the nematode experiments (acceptance threshold 5%). For the mouse experiments, we conducted this test between treatments and the number of mouse deaths or between strains and controls. For tests on nematodes, the experiment was repeated five times for *N. cyriacigeorgica* GUH-2 and *N. farcinica* 10152 to validate the test. The other strains (Table 1) were tested twice or three times each.

**Figure 2:**
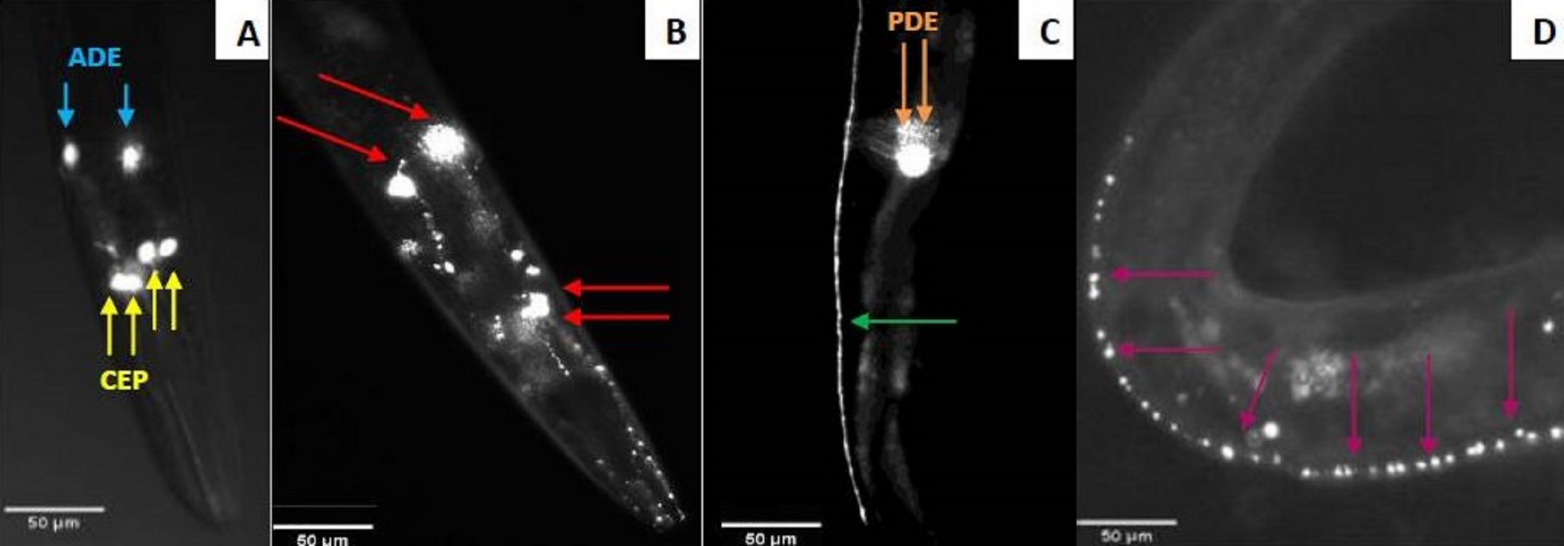
Fluorescent microscopy observation of *C. elegans* dopaminergic neurons. (A) Head of *C. elegans* exposed to control supernatant with unaltered neurons. Yellow arrows indicate the four CEP neurons and the blue ones indicate the two ADE neurons. (B) Damaged head of *C. elegans*. The red arrows show four neurons (2 ADE and 2 CEP) still present and the axons had blebbing. Two CEP neurons showed no visible fluorescence. Nematodes exposed to * N. cyriacigeorgica* supernatant were used for this picture. (C) The dendrites of dopaminergic neuron posterior (PDE) *C. elegans* exposed to control supernatant. (D) Dendrites of posterior dopaminergic neurons (PDE) with blebbing characterized by the appearance of visible dots along the axon. Nematode exposed to *N. farcinica* supernatant was used for this picture. Worms were observed through a X20 lens.

## Results

### Bacterial induction of dopaminergic neurodegeneration

**Figure 3:**
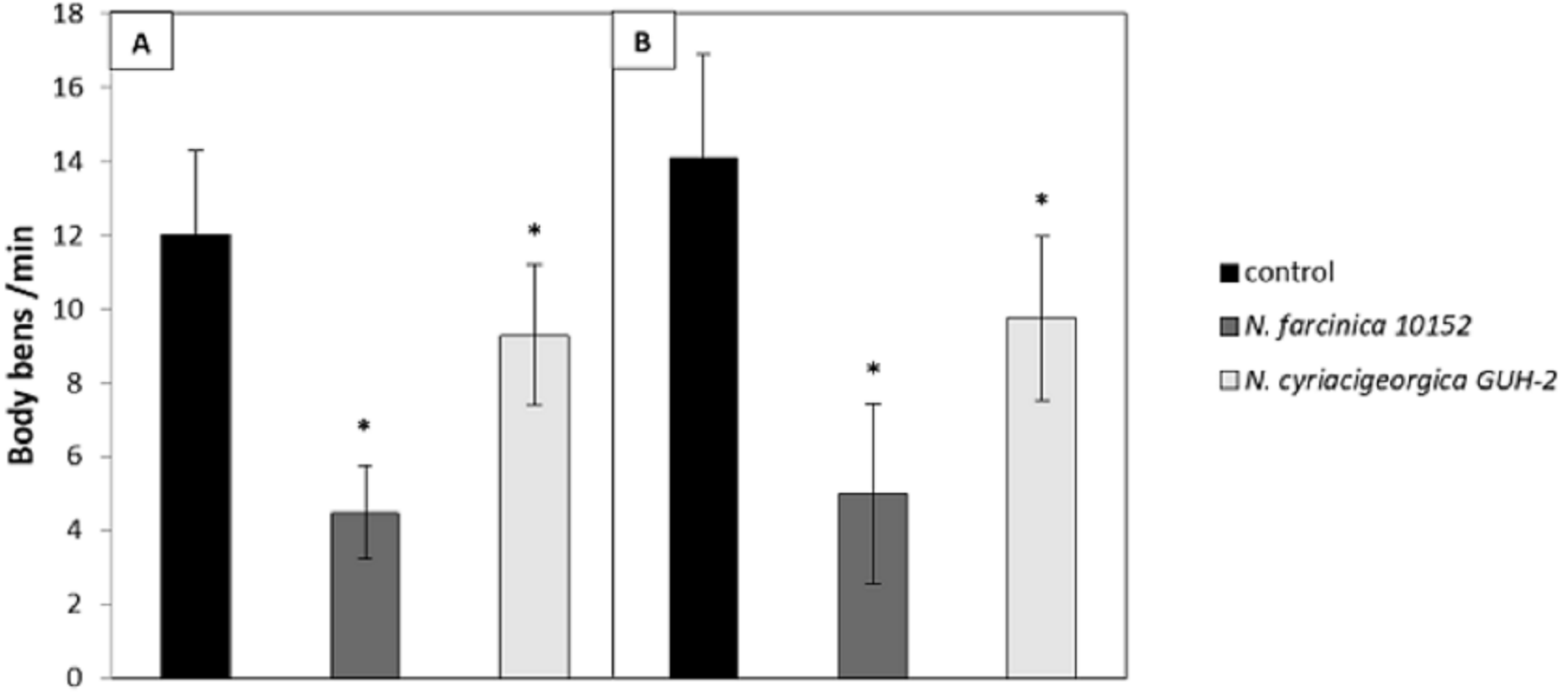
Effect of supernatants on *C. elegans* locomotion. (A) Worms of the wild-type strains N2 from synchronized eggs were raised in the presence or absence (control) of bacterial supernatants. (B) Worms of the transgenic strain BY250 with GFP expression from synchronized eggs were raised in the presence or absence (control) of bacterial supernatants. The locomotion of each worm was examined by counting the number of body-bends per min (*n* = 20∕treatment). Data are presented as the mean ± SD. ^∗^*p* < 0.05.

The neurotoxicity of metabolites excreted by *N. cyriacigeorgica, N. asteroides* and *N. farcinica* (Bennett medium culture) was tested on the nematode *C. elegans* targeted with GFP on dopaminergic neurons receptors. When the nematodes were exposed to bacterial supernatant for 10 days, dendrite blebbing, neuronal cell body rounding, or cell body and/or process loss were monitored. Deformed neurons and blebbing processes were repeatedly monitored, but neuronal loss seldom occurred (Fig. 2). Significant effects on the degeneration of *C. elegans* dopaminergic neurons (*p* < 0.05) were observed for *N. cyriacigeorgica* GUH-2, *N. cyriacigeorgica* N27, *N. cyriacigeorgica* 04.100 and *N. farcinica* IFM 10152 culture supernatants (Table 2). For *N. cyriacigeorgica* GUH-2, 36.7% (11/30) nematodes were affected: 82% showed dendrite blebbing, 73% neuronal cell body rounding and 9% neuronal loss. For *N. cyriacigeorgica* N27, 33.3% (10/30) nematodes were affected and, among them, 90% had dendrite blebbing and 50% showed neuronal cell rounding. For *N. farcinica* IFM 10152, 53% (17/32) nematodes were affected: 70.5% of these showed dendrite blebbing, 70.5% neuronal cell rounding, and 23.5% neuronal cell loss (Table 2). Fisher exact tests indicated that the findings for two strains were close to significance: *N. cyriacigeorgica* 04.100, with 30% (9/30) of nematodes affected and *N. asteroides* ATCC19247 with 25.8% (8/31) of nematodes affected. Taking the overall populations into account, we could not draw clear conclusions for both strains, but the marked difference in the significance levels obtained for these two strains indicated that *N. cyriacigeorgica* 04.100 had an effect on neurons (*p* = 0.042), while *N. asteroides* ATCC19247 had no effect (*p* = 0.082). We also performed a behavioral test for the dopamine function using a nematode touch sensitivity test; firstly to ensure that the nematodes were still alive and, secondly, to detect dopamine function alterations. *N. farcinica* 10152 or *N. cyriacigeorgica* GUH-2 strains induced higher neurodegeneration (Table 2) and, for these strains, we observed nematode behavioral disorders. The control nematodes (N2 and BY250) had functional dopaminergic neurons and the touch responses included receding movement followed by rapid forward leak. When nematodes were in contact with supernatant from *N. farcinica* 10152 or *N. cyriacigeorgica* GUH-2, we noted same behaviors as those observed without supernatants, but the nematode movements were very slow or only backwards. We also observed new behaviors: saccadic forward and backward movements without forward leak or motionless nematodes with only nose movements (Table S1). We performed a second test to quantify the behavioral phenotypes for dopaminergic functions. This test consisted of counting nematode body-bends per minute (Liu et al., 2015). For the controls (N2 and BY250) without supernatant, we counted 12 body-bends/min for N2 (WT strain) and 14.1 body-bends/min for BY250 (transgenic worms with GFP expression). Regardless of the nematode strain tested, worms had decreased mobility with all supernatants tested (4.5 and 9.4 body-bends/min for *N. farcinica* 10152 and *N. cyriacigeorgica* GUH-2 with *C. elegans* N2 and 5 and 9.75 body-bends/min for *N. farcinica* 10152 and *N. cyriacigeorgica* GUH-2 with *C. elegans* BY250). For both nematode lines, the supernatants had significant effects (Fig. 3).

**Table 2:** Summary of nervous system damage observed in 242 worms infected with various *Nocardia* supernatants in Bennett medium at 10 days. The percentages of affected *C. elegans* nematodes correspond to the number of nematodes having at least one dopaminergic neuron altered out of about 30 worms analyzed by fluorescence microscopy. Neuronal alteration was measured after 10 days of supernatantnematode exposure. Nervous system damage was observed by fluorescence microscopy and can be summarized as: (i) blebbing, (ii) cell body rounding, and (iii) loss of neuronal bodies. Each strain was statistically compared with the negative control via the Fisher exact test.

Strains	Number of nematodes	Number of nematodes with damage to the nervous system			
		Blebbing	Cell body rounding	Neuronal body process loss	Total
Nematode culture control	30	1 (3.33%)	0 (0%)	1 (3.33%)	1 (3.33%)
Medium culture control	29	2 (6.9%)	1 (3.45%)	1 (3.45%)	2 (6.9%)
*N. cyriacigeorgica* DSM 44484	30	4 (13.33%)	2 (6.67%)	1 (3.33%)	4 (13.33%)
*N. cyriacigeorgica* 04.107	30	5 (16.67%)	0 (0%)	0 (0%)	5 (16.67%)
*N. asteroides* ATCC19247	31	8 (25.81%)	2 (6.45%)	0 (0%)	8 (25.81%)
*N. cyriacigeorgica* 04.100	30	7 (23.33%)	2 (6.67%)	0 (0%)	9 (30%)^*^
*N. cyriacigeorgica* N27	30	9 (30%)	5 (16.67%)	0 (0%)	10 (33.33%)^*^
*N. cyriacigeorgica* GUH-2	30	9 (30%)	8 (26.67%)	1 (3.33%)	11 (36.67%)^*^
*N. farcinica* IFM 10,152	32	12 (37.5%)	12 (37.5%)	4 (12.5%)	17 (53.13%)^*^

**Notes.**

* *p* < 0.05.

### Mouse behavioral disorders induced by *Nocardia*

Mice were infected with a sublethal bacterial suspension (Beaman & Beaman, 1994). Three *Nocardia* species were tested, i.e., two clinical strains of *N. cyriacigeorgica*, one clinical strain of *N. farcinica*, and one environmental strain of *N. asteroides* (Table 1). The non-virulent status of *N. asteroides* 19247 defined by Beaman (1996) and Beaman & Beaman (1998) (was confirmed in this study (Table 3). The other strains induced behavioral disorders from day 6 post-infection (Table 3). Indeed, the number of mice with such disorders (and their intensity) increased until day 13 post-infection. These disorders were due to muscular rigidity and hemiparesis. The latter disorder was essentially visible by the position of the head, which was falling on one side. These damaged mice tended to turn in the same direction and begin to turn quickly when they were held by the tail. We also observed whole body tremors in some mice. Rhythmical and vertical movements of the head were also observed. These movements occurred more than 50 times in 30 s (Table S1), and they were very characteristic and different from control mouse movements. Mice infection with *N. farcinica* 10152 had more severe symptoms than those infected with *N. cyriacigeorgica*. Indeed, 45% of the mice (9/20) showed behavioral disorders after injection. A lethal dose (around 10^7^ CFU) of *N. cyriacigeorgica* GUH-2 was tested, which led to 50% mortality within 5 days post-injection.

**Table 3:** Summary of behavioral disorders observed in 103 mice infected with different *Nocardia* strains. Total affected mice correspond to the number of mice having at least one behavioral anomaly out of the 20 mice analyzed for each bacterial strain. Behavior anomalies were observed in mice after 13 days of infection and can be summarized by: (i) hemiparesis, (ii) vertical movement of the head, (iii) hemiparesis and trembling of the body, (iv) rigidity of movement, (v) death. The number of mice with abnormal behavior was indicated.

Strains	Dose	Number of mice	Number of deaths	Number of mice with neuronal anomalies^a^				
				Hemiparesis	Vertical movement of the head	Hemiparesis and body trembling	Rigidity of movement	Total
Medium culture control	–	6	0	0	0	0	0	0
*N asteroides* 19247	Sub-lethal	17	0	0	0	0	0	0
*N. farcinica* 10152	Sub-lethal	20	0	4	1	4	0	9
*N. cyriacigeorgica* 44484	Sub-lethal	20	0	2	3	0	2	7
*N. cyriacigeorgica* GUH-2	Sub-lethal	20	0	2	0	0	0	3
*N. cyriacigeorgica* GUH-2	Lethal	20	13	1	2	0	0	3

**Notes.**

a Total column corresponds to the affected number of mice having at least one behavioral disorder.

### Histology

**Figure 4:**
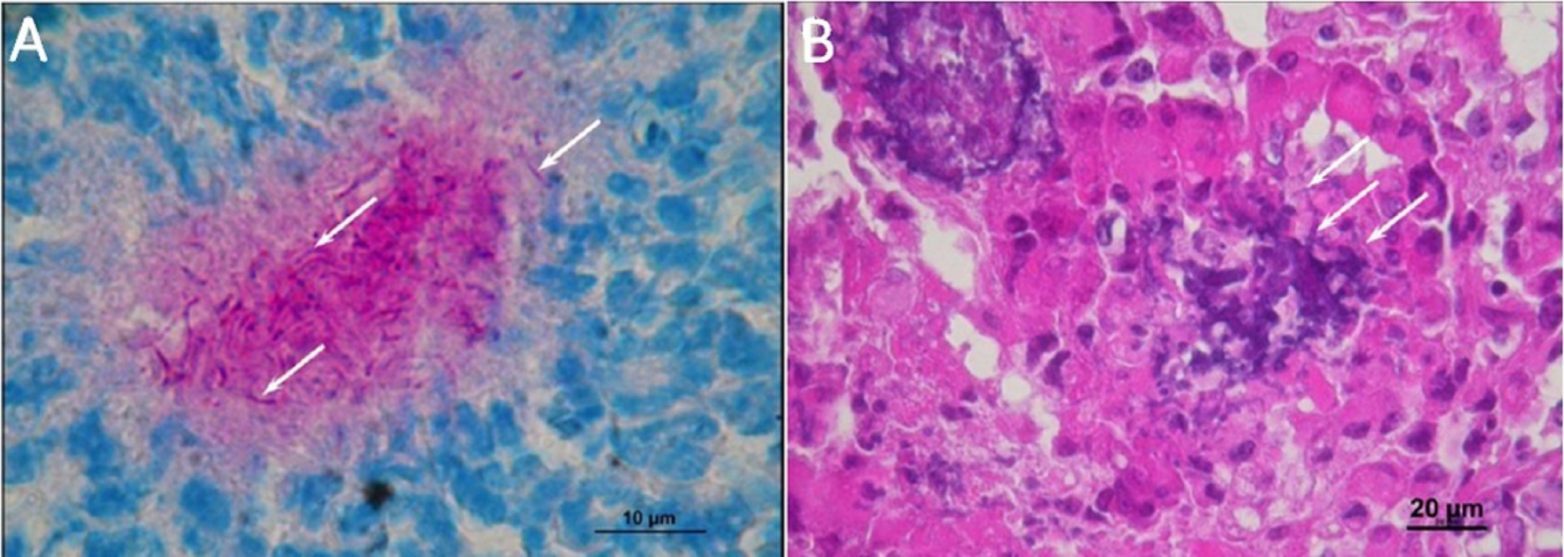
Histological observations on the mice that had died of sepsis after infection by *N. cyriacigeorgica* GUH-2. Arrows indicate the presence of *Nocardia*. (A) Staining Fite on a kidney, *Nocardia* appears to multiply in a localized manner. (B) Hematoxylin and eosin staining of a kidney localizing *Nocardia* development.

Necropsies for organ histological analysis were performed on mice that received a lethal injection of *N. cyriacigeorgica* GUH-2. Macroscopic observations revealed the presence of soft beige nodules on the spleen, kidney, myocardium, brain, liver and lung tissues. The organ histological findings revealed the presence of infectious foci. The largest lesions affected the kidney, spleen and myocardium. Lesions were characterized by abscesses, larger concentrations of inflammatory cells (poly- and mono-nuclear) and diffuse infiltration of these cells in the interstitial tissues. The kidney histological findings revealed the presence of filamentous bacteria strongly evocative of *Nocardia* (Fig. 4). These observations confirmed the dissemination of *Nocardia* throughout the body.

**Figure 5:**
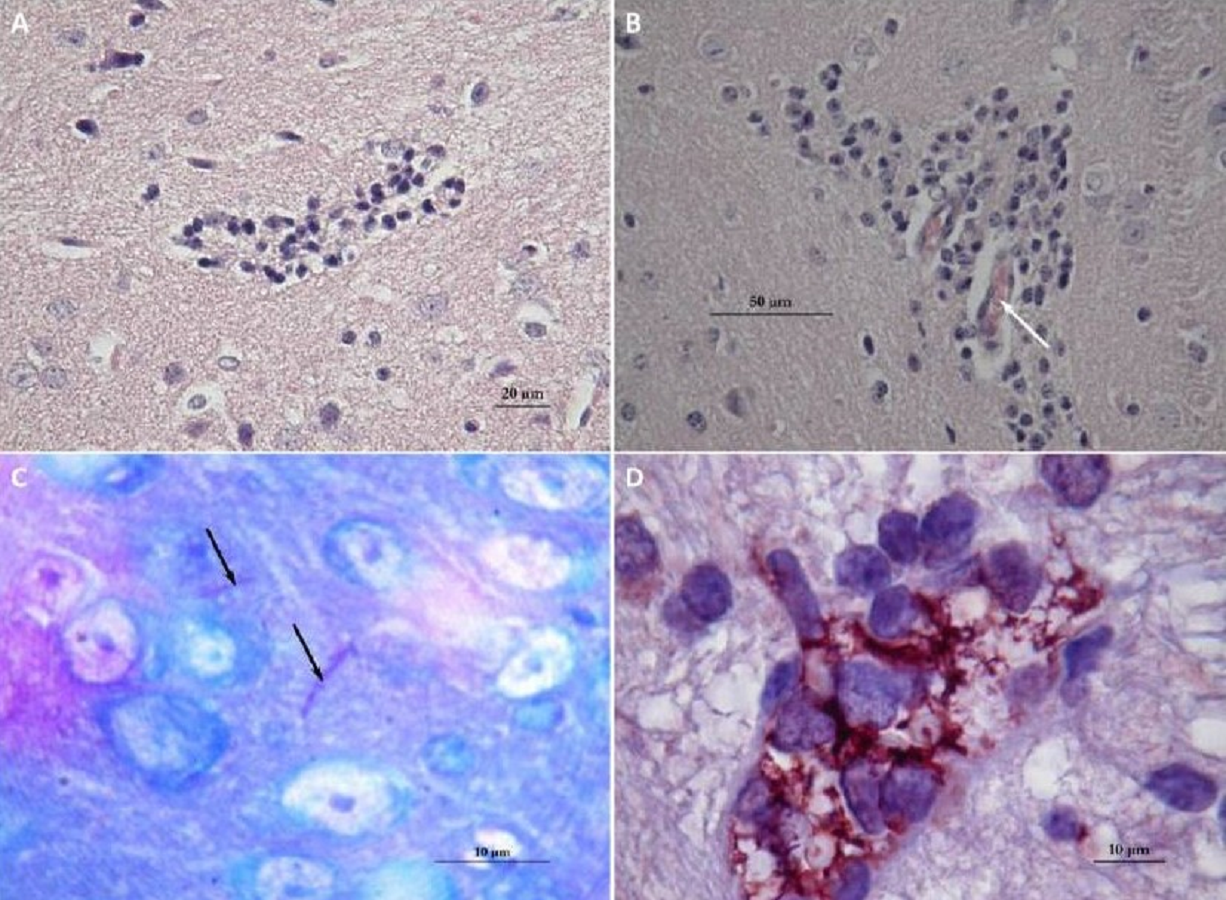
Histology of mice brains infected by *N. farcinica* 10152, with motor behavior disorders. (A) Hematoxylin-eosin showing a focus of gliosis at the base of the forebrain in mice with rhythmic vertical movements of the head and hemiparesis. (B–D) Observations on mice brains with only hemiparesis. (B) Hematoxylin-eosin staining showing lymphocytic sleeves around capillaries (white arrow). (C) Fite staining showing the presence of *Nocardia* cells (black arrows) in the middle of apparently healthy neurons. (D) Immunohistochemical analysis revealed the presence of *Nocardia* antigens (brick red) surrounded by microglial cells.

Brains of mice that had received a sub-lethal injection of *N. cyriacigeorgica* GUH-2 were recovered and analyzed. Analysis of sagittal brain slices revealed the presence of lesions of the gliosis cluster located at the bottom middle part of the telencephalon. An encephalon of a mouse infected with *N. cyriacigeorgica* GUH-2 but without motor symptoms revealed the presence of little gliosis clusters at the base of telencephalon with Harris-eosin hematoxylin staining (data not shown). There was slight inflammation at the base of cerebral hemispheres, but *Nocardia* was not revealed by staining (Fite, Gram, histochemical and immunochemical staining). Observations on a brain recovered from a mouse presenting motor symptoms (infected by strain *N. cyriacigeorgica* GUH-2) (Video S1) showed the presence of a diffuse gliosis at the base of the telencephalon and a small perivascular lymphocytic sleeve in the *medulla oblongata*. A little gliosis cluster was seen at the base of the telencephalon and one hyperchromatosis of neurons in the *medulla oblongata* (data not shown). The brains of mice with behavioral disorders (infected by *N. farcinica* 10152) showed a gliosis cluster at the base of the telencephalon, with Harris-eosin hematoxylin staining (Fig. 5A). The encephalon of one mouse showing hemiparesis, after infection with *N. farcinica* 10152 showed, by Harris-eosin hematoxylin staining, three gliosis clusters, one on the diencephalon and two at the base of the telencephalon, (Fig. 5B). Fite staining revealed the presence of *Nocardia*-like cells (Fig. 5C). Histochemical and immunochemical staining highlighted *Nocardia*-like cells in the cerebellum and in the *medulla oblongata* (Fig. 5D). It is noteworthy that at five weeks post-inoculation, *Nocardia*-like cells were only observed in mice with hemiparesis.

## Discussion

*Nocardia* strains were found to induce behavioral changes in mice, and some of their excreted metabolites could cause neuronal degeneration in the nematode *C. elegans*. Our data suggests that the transgenic strain BY250 *vtIs* 7 [Pdat-1:GFP] could be useful for investigating chemically-induced neurodegeneration. This nematode line allowed the detection of *Nocardia* strains producing secondary metabolite(s) in the broth, which may induce brain damage. This led to the first observation of a *N. farcinica* strain inducing behavioral disorders in mice. These results indicate that the ability to induce neurodegeneration could be widely distributed in the *Nocardia* genus.

### Dopaminergic neuron neurodegeneration

The *N. cyriacigeorgica* GUH-2 strain can invade the neuronal central system and cause dopaminergic neurodegeneration in mice (Ogata & Beaman, 1992). Here we demonstrate that this property induced by *N. cyriacigeorgica* can be obtained using a rapid test with the *C. elegans* BY250 *vtIs* 7 [Pdat-1:GFP]) line. This test, that involved exposing *Nocardia* supernatants to nematodes, highlighted damage on dopaminergic neurons. Supernatants were used because we hypothesized that dopaminergic neurodegeneration was due to metabolic compounds secreted by these bacteria. Thus, nematodes exposed to supernatants allowed us to test for the presence of metabolites involved in bacterial virulence. It is well known that pathogenesis may be connected to excreted metabolites among Actinobacteria. For example, nocobactine, a siderophore, was found to contribute to the cytotoxicity of *N. farcinica* 10152 (Hoshino et al., 2011; Ishikawa et al., 2004). The same was noted with mycobactin, a *Mycobacterium tuberculosis* siderophore (Krithika et al., 2006). These two siderophores are products of secondary metabolism. *Nocardia* is known to produce some of these virulence factors. For example, *N. cyriacigeorgica* GUH-2 supernatants have apoptotic activity on PC12 culture cells with inhibition of the three enzymatic activities of PC12 proteasomes and inhibition of only two of them for human proteasomes (Barry & Beaman, 2007). The major interest of this nematode test is the possibility of screening a large number of bacterial strains for their neurodegenerative potential before, or instead, of using mammalian models. In this study, seven *Nocardia* strains of environmental and clinical origin were tested. The results showed the ability of four *N. cyriacigeorgica* strains to significantly damage the neuronal system, including *N. cyriacigeorgica* 44484, which induced neuronal body loss but not significantly. This was probably due to a low number of observed nematodes. The statistical analysis findings would likely be stronger if we had increased the number of worms tested. This property did not seem to be restricted to the *N. cyriacigeorgica* GUH-2 strain as we had previously thought. In fact, the *N. cyriacigeorgica* N27 strain produced secondary metabolites that could substantially damage dopaminergic neurons. *N. farcinica* IFM 10152 had the same effect on nematodes. These excreted metabolites involved in virulence were detected in broths from clinical (i.e., GUH2, IFM 10152, 04.100) and environmental strains (i.e., N27). Human exposure to virulent *Nocardia* mainly occurs through contact with environmental matrices where this bacterium is present. This test thus confirmed the health hazards associated with environmental strains. However, the distribution of such metabolites involved in virulence among the various *Nocardia* species remains to be explored. Supernatants of non-virulent strains did not lead to neuronal degeneracy.

The *N. cyriacigeorgica* N27 strain was isolated from a hydrocarbon-contaminated environment (OFN (Observatoire Français des Nocardioses), 2000, unpublished data). Environmental exposure to such a pathogen is possible for populations in contact with highly hydrocarbon contaminated environments such as urban areas. More environmental *Nocardia* species could likely induce the same symptoms and this needs to be further explored. This test will be applied to assess a larger panel of species and strains. Neuronal damage induction is not exclusive to *Nocardia* and can be found in other bacterial genera such as *Streptomyces* (Caldwell et al., 2009). Caldwell et al. (2009) showed that *S. venezuelae* could induce effects neurons similar to those observed with *Nocardia* secreted metabolites. After testing the potential of different *Streptomyces* strains to induce dopaminergic neuron degeneration in *C. elegans*, *S. venezuelae* was found to have a significant effect on nematodes after four days of exposure to the culture supernatant. Nematodes in contact with supernatants had damaged neurons that were deformed and showed blebbings, as also noted in our study (Caldwell et al., 2009). It is well known that blebbing frequency appearance can increase with age of nematode but these aged neurons are not undergoing apoptosis or necrosis (Chew et al., 2013). All experiments were carried out in comparison with controls (Table 2). Only one or two nematodes had neuronal structure modifications out the 30 nematodes tested. These neuronal anomalies were likely due to the nematode age, for the other ones we did not have issues with the nematode age. We took account of the controls in our statistical analyses (Table 2). We considered the possible decrease in fluorescence when using GFP. However, if our results had been partially due to a decrease in GFP expression, we would have also observed a loss of fluorescence along the axon. In our experiments, as we retained fluorescence along the axon for the controls and tests, we conclude that the results were not due to decreased of GFP expression. These results were confirmed by the findings of the two behavioral tests performed and the use of wild-type and transgenic nematode strains. We observed modifications in nematode behavior related to dopaminergic neurons, like movements induced by a touch sensitivity test and the mobility (body-bends) of the worms. We obtained the same results with both nematode strains (N2 and BY250), so we conclude that the observed effect was due to the bacterial supernatant. In our experiment, all nematodes were of the same age because we selected nematodes at the L4 development stage, so the differences observed between strains must have been due to the secreted metabolites. Regarding the number of nematodes affected and the severity of the induced disorders, metabolites from *N. farcinica* 10152 had stronger neurotoxic effects than *N. cyriacigeorgica* GUH-2. In further analyses, a nematode with other neuronal GFP markers will be used to see if our results are specific to dopaminergic neurons or if metabolites secreted by *Nocardia* strains could affect other kinds of neurons.

### Behavioral disorders in mice and histology of encephala

*Nocardia* species which induced neurodegeneration in nematodes (including strains inducing neuronal body process loss) were tested in mice to confirm the onset of behavioral disorders in the mammalian model. The results obtained showed the implication of *Nocardia* strains in the onset of behavioral disorders in mice. Analyses of brain slices revealed lesions at the base of the telencephalon likely responsible for the observed responses in mice. These observations were generally in line with those of Kohbata & Beaman (1991). All strains tested led to significant difficulties for the mice to move forward, as shown in Beaman & Tam (2008), but they did not result in a vertical positioning of the tail (Kohbata & Shimokawa, 1993).

The histology of encephala showed the immune response of the infection (gliosis, lymphocytes) but did not reveal the presence of *Nocardia* cells in mice with rhythmical and vertical movements of the head, as observed by Beaman & Tam (2008) and Kohbata & Beaman (1991) (Fig. 5). *Nocardia* cells were observed in brain of mice that had undergone hemiparesis but also in kidney cells of mice that died of septicemia. These results revealed that new *Nocardia* strains could be responsible for mouse behavioral disorders (*N. farcinica* 10152 and *N cyriacigeorgica* 44484). This is the first time that *N. farcinica* was shown to be involved in movement disorders and detected among mouse brain tissues. Sequencing of the *N. farcinica* IFM10152 genome revealed the presence of virulence genes, such as Mce proteins (mammalian cell entry protein), antigen 85 family proteins, superoxide dismutase and factors involved in adhesion and invasion of host cells, as noted in the *N. cyriacigeorgica* GUH-2 genome (Zoropogui et al., 2013). These proteins could be involved in the ability of *N. farcinica* to induce neuronal degeneration and this hypothesis needs to be further explored (Ishikawa et al., 2004).

The mouse experiment results confirmed those obtained with nematodes. They confirmed that *N. farcinica* 10152 was more virulent than *N. cyriacigeorgica* GUH-2 according to the severity of the disorders observed. *N. cyriacigeorgica* 44484 induced symptoms that could be associated to neurodegeneration in mouse experiments (Table 3), but not significantly in nematode tests, even though we showed one neuronal body process loss. This difference could be related to a lower level of production of the secondary metabolites involved in the neurodegeneration of dopaminergic neurons with this strain. The different culture time for *Nocardia* obtained with preliminary tests confirmed that *Nocardia* species produce neurotoxic compounds at different rates. The results obtained with *N. cyriacigeorgica* 44484 were important because they showed that *C. elegans* could be used in pre-screening tests before performing mouse experiments, provided that neuronal body process loss is taken into account. This difference between results in mice and nematode with this strain indicates the need to take into account the growth rate precisely and the ODs which are parameters difficult to control in Actinobacteria.

## Conclusion

The aim of this study was to develop a method to investigate *Nocardia* properties involved in neuronal virulence and assess the health hazards of *Nocardia* strains. We thus used the *C. elegans* BY250 *vtIs* 7 [Pdat-1:GFP]) line as a model system, and it seems to be a relevant model for studying neuronal dopaminergic damage, as suggested previously (Ali & Rajini, 2012; Harrington et al., 2010; Vistbakka et al., 2012).

In mice, we tested strains affecting dopaminergic neurons of nematodes, including those inducing neuronal body process losses. This experiment revealed the ability of the bacteria to induce behavioral disorders in the host animal while affecting neurological areas. Our results confirmed those obtained by Kohbata & Beaman (1991) and Beaman & Tam (2008).

Our study revealed that *N. cyriacigeorgica* (not only the GUH-2 strain) and *N. farcinica* could induce dopaminergic neuron degeneration in *C. elegans* and induce behavioral disorders that may be related to neuronal damage in mice, despite their origins. In the light of our results, *N. farcinica* 10152 seems to have had a greater neurotoxic effect on dopaminergic neurons than other tested strains. Tests on the *C. elegans* BY250 *vtIs* 7 [Pdat-1:GFP]) line appeared to be faster and easier to perform than the mouse experiments for detecting neurodegeneration, and this is a good model to screen numerous bacteria. This nematode test could be a good model for bioactivity guided research on bioactive bacterial compounds to find the molecule(s) responsible for dopaminergic neurodegeneration.

##  Supplemental Information

10.7717/peerj.3823/supp-1 Video S1 Mice infected by *N. cyriacigeorgica* GUH-2 presenting motor symptoms Behavioral disorders in two mice. One with the rhythmic and vertical movements of the head (mouse up) and the other with hemiparesis (mouse down).

10.7717/peerj.3823/supp-2 Supplemental Information 1 Number of nematodes affected in their movement using the touch response test Wild type response to the touch response test is characterised by backward movements of the nematode and then a leak forward. We compared this reaction on N2 and BY250 nematodes lineages that were not in contact with supernatant (control). Nematode affected had the same reactions following the touching but with slow reactions or only backwards. We observed also forwards/backwards movements or motionless nematodes with only a movement of the nose. These kinds of phenotypes were observed on nematodes that were in contact with bacterial supernatant (*N.farcinica* 10152 or *N.cyriacigeorgica* GUH-2).

10.7717/peerj.3823/supp-3 Data S1 Raw data—Fig. 1

10.7717/peerj.3823/supp-4 Data S2 Raw data—Fig. 2

10.7717/peerj.3823/supp-5 Data S3 Raw data—Fig. 3

## Additional Information and Declarations

### Data Availability

The following information was supplied regarding data availability: The raw data has been uploaded as Supplemental Files.
