# Supplementary material for: Virulence test using nematodes to prescreen Nocardia species capable of inducing neurodegeneration and behavioral disorders
Source: PeerJ. 2017 Oct 10;5:e3823. doi: 10.7717/peerj.3823 (PMC5639870; doi:10.7717/peerj.3823)
Supplement: Supplemental Information 1 — Wild type response to the touch response test is characterised by backward movements of the nematode and then a leak forward. We compared this reaction on N2 and BY250 nematodes lineages that were not in contact with supernatant (control). Nematode affected had the same reactions following the touching but with slow reactions or only backwards. We observed also forwards/backwards movements or motionless nematodes with only a movement of the nose. These kinds of phenotypes were observed on nematodes that were in contact with bacterial supernatant (N.farcinica 10152 or N.cyriacigeorgica GUH-2). [file peerj-05-3823-s002.pdf]

Strains	Non-affected	Affected				
		Slow reactions	backwards movements	forward/backward movements	Motionless nematodes	Total
N2.control	30	0	0	0	0	0
N. farcinica	4	20	12	4	0	26*
N. cyriacigeorgica	8	20	14	1	1	22*
BY250.control	30	0	0	0	0	0
N. farcinica	3	5	4	0	1	7*
N. cyriacigeorgica	5	12	15	2	0	25*
